# Population genetic structure and phenotypic diversity of *Aspidodera raillieti* (Nematoda: Heterakoidea), a parasite of Didelphini marsupials in Brazil’s South and Southeast Atlantic Forest

**DOI:** 10.1186/s13071-022-05288-6

**Published:** 2022-06-13

**Authors:** Karina Varella, Roberto do Val Vilela, Rosana Gentile, Thiago dos Santos Cardoso, Sócrates Fraga da Costa-Neto, Arnaldo Maldonado Júnior

**Affiliations:** 1 Programa de Pós-Graduação em Biologia Parasitária (PPGBP), Instituto Oswaldo Cruz, Fundação Oswaldo Cruz, Manguinhos, Rio de Janeiro, RJ Brazil; 2 Laboratório de Biologia e Parasitologia de Mamíferos Silvestres Reservatórios, Instituto Oswaldo Cruz, Fundação Oswaldo Cruz, Manguinhos, Rio de Janeiro, RJ Brazil; 3 Fiocruz Mata Atlântica, Fundação Oswaldo Cruz, Rio de Janeiro, RJ Brazil

**Keywords:** Aspidoderidae, *Didelphis*, *Philander*, AMOVA, Fixation index, Mantel test, Morphology, MT-CO1, Phylogeny, Phylogeography

## Abstract

**Background:**

The population genetics of parasites may be influenced by host specificity, life cycle, host geographical range, evolutionary history, and host population structure. The nematode *Aspidodera raillieti* infects different marsupial and rodent hosts in the Nearctic and Neotropical regions, implying a gene flow among populations. However, niche diversification of the main hosts of *A. raillieti* in superimposed areas may provide conditions for population genetic structuring within this parasite species. We examined the genetic structuring of *A. raillieti* infecting three marsupial species co-occurring along the South and Southeast Brazilian Atlantic Forest, a hotspot of biodiversity.

**Methods:**

We employed morphometric analyses and partial mitochondrial cytochrome c oxidase I gene sequences (MT-CO1) to characterize populations via phylogenetic and phylogeographic analyses.

**Results:**

Among 175 *A. raillieti* specimens recovered from the marsupial hosts *Didelphis aurita*, *D. albiventris*, and *Philander quica*, we identified 99 MT-CO1 haplotypes forming four haplogroups and four clades in networks and phylogenetic trees, respectively. Clades I and II encompassed parasites of *D. albiventris* from the South region, clade III comprised parasites of *D. aurita* from the South and Southeast regions, and clade IV encompassed parasites of *D. aurita* and *D. albiventris* from the South and Southeast regions and parasites of *P. quica* from the South region. High genetic differentiation between clades, with a high fixation index and greater genetic variation in the analysis of molecular variance (AMOVA), indicated low gene flow between clades. Haplotypes shared among host species revealed a lack of host specificity. A significant correlation in the Mantel test suggested parasite isolation by distance, while there was no evidence of geographical structure between populations. Negative neutrality test values for clades III and IV suggested recent population expansion. Morphometric differentiation between *A. raillieti* specimens recovered from different host species, as well as from different localities, was more evident in males.

**Conclusion:**

The genetic structure of *A. raillieti* populations in the South and Southeast Atlantic Forest resulted from historical events rather than from current geographical distribution or host specificity. We also demonstrate morphometric variation associated with host species and localities, suggesting phenotypic plasticity to host attributes and to spatial variables.

**Graphical Abstract:**

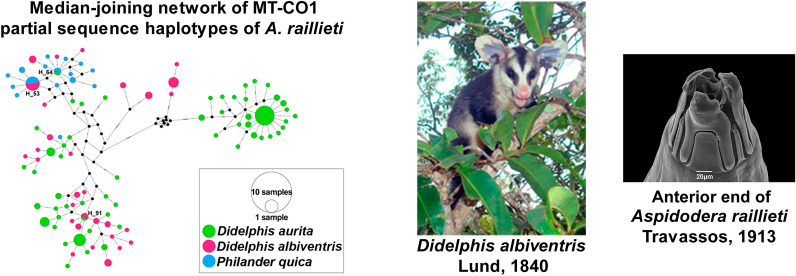

**Supplementary Information:**

The online version contains supplementary material available at 10.1186/s13071-022-05288-6.

## Background

The nematodes of the family Aspidoderidae Freitas, 1956, superfamily Heterakoidea Railliet & Henry, 1912, comprise *Aspidodera* Railliet & Henry, 1912; *Paraspidodera* Travassos, 1914; *Lauroia* Proença, 1938; and *Nematomystes* Sutton, Durette-Desset & Chabaud, 1980 [1–3]. The genus *Aspidodera* is morphologically characterized by cuticular expansion forming a cephalic hood with cordons arranged in six longitudinal loops at the anterior end, three lips, an oesophagus with a terminal bulb, a ventral sucker on males, a pair of spicules and a gubernaculum, and a posterior region ending in a digitiform projection [4, 5]. *Aspidodera* spp. are distributed along the southern Nearctic and the entire Neotropical region, parasitizing the cecum and the large intestines of mammals of the orders Didelphimorphia, Cingulata, Pilosa, and Rodentia [6].

*Aspidodera raillieti* Travassos, 1913 [7] is widely distributed in the Americas, occurring in the USA, Mexico, Guatemala, Costa Rica, Panama, Trinidad and Tobago, Colombia, Suriname, French Guiana, Brazil, Bolivia, Paraguay, and Argentina [2, 4, 5, 8]. Some species of *Aspidodera* were considered junior synonyms of *A. raillieti* because the morphological and morphometric differences used to propose these new species are not diagnostic characters and thus do not allow distinguishing them from *A. raillieti*. Among them are *A. harwoodi* Chandler, 1932 described from *D. virginiana* Kerr, 1792 in the USA; *A. diazungriai* Masf-Pallares and Vergara, 1971, parasitizing *Didelphis pernigra* J. A. Allen, 1900 in Paraguay; and *A. vicentei* Kohn, Fernandes and Mello, 1982, described from *Nectomys squamipes* Brants, 1827 in Brazil [5]. These findings suggest that the morphological and morphometric variability of *A. raillieti* might be associated with its geographical distribution and its hosts.

*Aspidodera raillieti* has been found in all Brazilian biomes in areas of Amazonia, Atlantic Forest, Caatinga, Cerrado, Pampa, and Pantanal. This nematode was found parasitizing different species of marsupials, including *Didelphis aurita* Wied-Neuwied, 1826; *Didelphis albiventris* Lund, 1840; *Didelphis marsupialis* Linnaeus, 1758; *Philander quica* (Temminck, 1824); *Philander opossum* (Linnaeus, 1758); *Chironectes minimus* (Zimmermann, 1780); *Metachirus myosurus* (É. Geoffroy St.-Hilaire, 1803); *Caluromys lanatus* (Olfers, 1818); *Marmosops incanus* (Lund, 1840); and a single rodent species, *Nectomys squamipes* (Brants, 1827) [9–11]. Four marsupial species of the tribe Didelphini occur in sympatry and are found infected by *A. raillieti* in areas of the Brazilian Atlantic Forest: *D. aurita*, *D. albiventris, P. quica*, and *C. minimus* [12]. The Atlantic Forest is among the most altered Brazilian biomes due to urban expansion and economic activities, retaining only approximately 12% of its original coverage [13–16].

Parasite populations may have their genetic structure influenced by several factors, such as the degree of host specificity, effective population size, geographical distance between populations, host dispersal ability, evolutionary history, host population structures, and life cycle complexity [16–19]. The low degree of host specificity exhibited by *A. raillieti* presupposes significant gene flow between populations of different host species, despite this nematode having a monoxenous life cycle [10]. Therefore, the *A. raillieti* population structure is expected to depend on host movements [17, 20–22], geographical distance, and/or historical processes [18].

A study on the ecological modelling of *D. aurita* and *D. albiventris*, based on climatic niches throughout their geographical distributions, showed that these marsupials might explore different niches in areas where they co-occur, mostly in areas of forest-grassland mosaics [23]. In addition, *D. aurita* may act as a biotic barrier for *D. albiventris,* where this habitat mosaic is not available for their coexistence [23]. These different host ecological characteristics may promote favourable conditions for the emergence of genetic structuring patterns in parasite populations, which is consistent with the use of different host species.

However, the distribution of *P. quica* overlaps with that of *D. aurita* and partially overlaps with that of *D. albiventris* [24, 25]. *Philander quica* and *D. aurita* have niche overlap and compete with each other [26, 27]; both species occur in humid forested areas [27], and predation upon *P. quica* by *D. aurita* occurs [27, 28], which may favour parasite transmission from one host species to another. These aspects may promote gene flow among the parasite populations of different hosts.

Nevertheless, another study demonstrated that populations of *D. albiventris* exhibit patterns of isolation by distance when comparing disjunction distributions between South and Southeast Brazil [29]. The same pattern was observed for *D. aurita* at a regional scale in Southeast Brazil [30, 31]. Likewise, this pattern may occur in parasite populations.

In this context, we aimed to examine the population genetic structure of the nematode *A. raillieti*, a parasite of the marsupials *D. aurita*, *D. albiventris*, and *P. quica* in South and Southeast Atlantic Forest localities. We hypothesized that *A. raillieti* populations are genetically structured as a function of their host species and/or geographical distances.

## Methods

### Host sampling

**Fig. 1:**
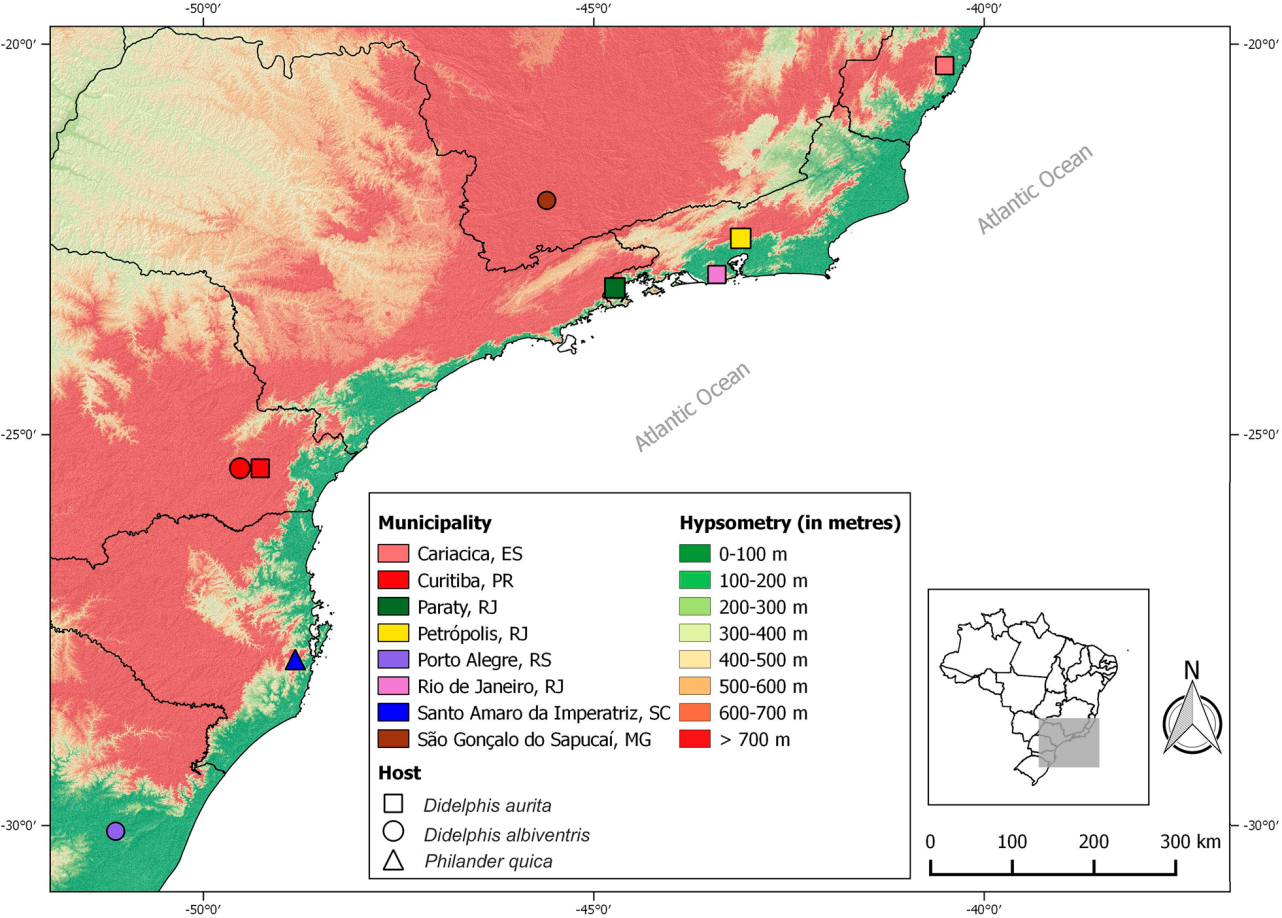
Sampling localities of *A. raillieti* recovered from marsupial hosts in southern and southeastern Brazil. The geometric shapes represent different host species, and shape colours indicate their respective municipalities. The background map displays elevation values in hypsometric tints

This study was conducted in eight localities in the Brazilian Atlantic Forest, from the state of Rio Grande do Sul in Brazil’s South Region to the state of Espírito Santo in the Southeast Region (Fig. 1, Tables 1 and 2). These localities include different natural forest formations, comprising dense ombrophilous forest, mixed ombrophilous forest, and semi-deciduous seasonal forest (Table 1) [14].

**Table 1:** Host species used in the present study, the total number of host individuals analysed, and specimens (*N*) per host species, with their respective locality abbreviations, municipalities and states, geographical coordinates, heights in metres above sea level (m.a.s.l.), and locality descriptions

Host species	*N* host euthanized	*N*	Locality	Municipality, state	Geographical coordinates	Height (m.a.s.l.)	Locality description
*D. aurita*	3	1	CAR-ES	Cariacica, Espírito Santo	20° 16′ 28″ S, 40° 30′ 25″ W	471	Duas Bocas Biological Reserve—Atlantic Forest area characterized by a submontane dense ombrophilous vegetation and areas with human activities (fishing and agriculture) within and around the reserve
*D. aurita*	12	3	PET-RJ	Petrópolis, Rio de Janeiro	22° 29′ 09″ S, 43° 07′ 11″ W	1102	Serra dos Órgãos National Park—a preserved forest area and one of the most important remnants of the Atlantic Forest in Brazil, characterized by a Montane Atlantic Forest of dense ombrophilous vegetation
*D. aurita*	5	4	RIO-RJ	Rio de Janeiro, Rio de Janeiro	22° 56′ 22″ S, 43° 24′ 14″ W	20	FIOCRUZ Atlantic Forest Biological Station—an urban–sylvatic interface environment including peridomicile and disturbed forest areas in the buffer zone of the Pedra Branca State Park, the largest forest reserve in an urban region in the Americas
*D. aurita*	27	7	PTY-RJ	Paraty, Rio de Janeiro	23° 07′ 15″ S, 44° 43′ 52″ W	123	Juatinga Ecological Reserve—a preserved forest area considered an important remnant of the Brazilian Atlantic Forest located on the southern coast of the state of Rio de Janeiro between mountain ranges
*D. albiventris*	4	2	SGS-MG	São Gonçalo do Sapucaí, Minas Gerais	21° 59′ 40″ S, 45° 36′ 26″ W	914	Ribeiros district—Atlantic Forest area characterized by montane seasonal semi-deciduous forest vegetation. Fragments of preserved primary and secondary vegetation immersed in an extensive anthropic matrix, in addition to areas with different types of agricultural and livestock production
*D. aurita*	10	2	CUR-PR	Curitiba, Paraná	25° 33′ 19″ S, 49° 1′ 06″ W	924	Barigui Park, Botanical Garden, Zoo—small fragments of altered primary vegetation or secondary vegetation of subtropical forests with araucaria and some preserved areas in and around the city
*D. albiventris*	11	3					
*P. quica*	11	4	SAI-SC	Santo Amaro da Imperatriz, Santa Catarina	27° 52′ 27″ S, 48° 48′ 52″ W	200	Serra do Tabuleiro State Park—areas of dense ombrophilous vegetation with continuous canopy cover, semi-open understorey, predominance of medium-sized trees, and presence of watercourses
*D. albiventris*	16	4	POA-RS	Porto Alegre, Rio Grande do Sul	30° 04′ 17″ S, 51° 07′ 28″ W	84	Vila Laranjeiras, Morro do Santana, Morro da Polícia and Campus UFRGS—peridomicile areas near forest fragments in the urban region of the city

**Table 2:** Distances between localities where hosts infected by *A. raillieti* were collected (in kilometres)

	CAR-ES	RIO-RJ	PET-RJ	PTY-RJ	SGS-MG	CUR-PR	SAI-SC
CAR-ES							
RIO-RJ	393						
PET-RJ	343	50					
PTY-RJ	524	145	186				
SGS-MG	584	253	279	131			
CUR-PR	1056	678	721	535	483		
SAI-SC	1102	710	759	581	570	185	
POA-RS	1402	1010	1059	879	855	389	300

Marsupials were captured using Tomahawk Live Trap (Hazelhurst, WI) model 201 traps (16″ × 5″ × 5″) baited with a mixture of peanut butter, banana, oats, and bacon.

### Helminth recovery

The digestive tract of marsupials was screened for parasites, and both the large intestine and the cecum were examined for the presence of specimens of *A. raillieti*. Organs were placed separately in Petri dishes, washed twice in physiological saline solution (NaCl 0.85%) to remove tissue debris, and stored in 70% ethanol solution. For examination, nematodes were clarified in 25% glycerin alcohol. Measurements and drawings were produced with the aid of a camera lucida attached to a Nikon Eclipse E200 MV R microscope. Specimens were randomly selected for measurements. The number of specimens used for morphometric analyses was based on the parasite burden (Table 3).

**Table 3:** Female, male, and total numbers of marsupial hosts and *A. raillieti* specimens measured from each locality and region

Region	Locality	Host species	Hosts			Helminths measured		
			♀	♂	Total	♀	♂	Total
Southeast	CAR-ES	*D. aurita*	–	1	1	1	1	2
	SGS-MG	*D. albiventris*	1	1	2	10	10	20
	RIO-RJ	*D. aurita*	2	2	4	20	19	39
	PTY-RJ		5	1	6	19	17	36
	PET-RJ		2	1	3	10	5	15
South	CUR-PR	*D. aurita*	2	–	2	3	10	13
		*D. albiventris*	1	1	2	10	14	24
	SAI-SC	*P. quica*	3	1	4	16	24	40
	POA-RS	*D. albiventris*	3	1	4	14	16	30

### Discriminant analysis of principal components

We performed a discriminant analysis of principal components (DAPC) [32] to compare helminth morphometric differences considering localities and host species (except for Cariacica, Espírito Santo [CAR-ES] due to the low sample size of hosts and helminths recovered).

DAPC is a robust method for describing variations between defined groups, selecting principal components (PCs) that explain the greatest variation between groups while minimizing the variation within each group. We used the cross-validation optimization procedure to identify the ideal number of PCs to be retained by DAPC and selected the components associated with the lowest root mean squared error [33]. Finally, we determined the percentage of *A. raillieti* specimens correctly classified within their original group. Thus, we used DAPC to evaluate the results from the genetic analyses. DAPC was performed using the package ‘adegenet’ [34] in the R software environment, version 4.0.2 [35].

### Genomic DNA isolation, amplification, and sequencing

We isolated genomic DNA from mid-section fragments of each adult specimen of *A. raillieti* using the QIAamp DNA Mini Kit (QIAGEN, Hilden, Germany) according to the manufacturer’s instructions. Before DNA isolation, each specimen was morphologically characterized and subsequently washed in distilled water. Amplifications of the mitochondrial cytochrome *c* oxidase subunit I gene (MT-CO1) by polymerase chain reaction (PCR) were performed individually for each *A. raillieti* specimen using the primer cocktail described by Prosser et al. [36]. Each reaction contained 12.5 μl of PCR Master Mix (Promega Corporation) (50 units/ml *Taq* DNA polymerase, 400 μM dATP, 400 μM dGTP, 400 μM dCTP, 400 μM dTTP, 3 mM MgCl_2_), 0.5 μl of each primer cocktail (10 μM of a three-forward primer mix and 10 μM of a three-reverse primer mix), 1 μl of genomic DNA, and ultrapure water in a total volume of 25 μl. Thermal cycling conditions were 94 °C for 1 min; five cycles at 94 °C for 40 s, 45 °C for 40 s, and 72 °C for 1 min; 35 cycles at 94 °C for 40 s, 51 °C for 40 s, and 72 °C for 1 min; and a final extension at 72 °C for 5 min [36]. Successfully amplified amplicons were purified using the Illustra GFX PCR DNA and Gel Band Purification Kit (GE Healthcare, Little Chalfont, Bucks, UK) following the manufacturer’s protocol and then cycle sequenced using the Big Dye Terminator v3.1 Cycle Sequencing Kit (Applied Biosystems, Carlsbad, CA, USA), individually for each cocktail primer for better accuracy. Cycle-sequenced product precipitation, formamide resuspension, and sequencing were conducted at the Capillary Sequencing (SANGER) Platform, P01-001-RPT/FIOCRUZ (https://plataformas.fiocruz.br/). Sequencing was performed using the 96-capillary 3730xl DNA Analyzer (Applied Biosystems).

### Molecular phylogenetic and phylogeographic analyses

For each specimen, DNA sequencing reads were assembled into contigs and edited for ambiguities using the Geneious version 9.1.8 bioinformatics software platform [37], resulting in a consensus sequence.

All sequences obtained in this study were deposited in GenBank (accession numbers OL676808–OL676982) (Additional file 1: Table S1). Two datasets were used in this study. The first dataset, used for phylogenetic inferences, included the sequences we generated and those available in GenBank (Additional file 1: Table S1), including sequences from other species of the family Aspidoderidae (Table 4). As an out-group, we added a sequence of *Lauroia trinidadensis* (Aspidoderidae). Since *Nematomystes* and *A. raillieti* formed a monophyletic group in previous studies [2, 8], we included *Nematomystes* spp. to the ingroup in our phylogenetic analyses. The second dataset, used for phylogeographic analyses, included only our 175 sequences of *A. raillieti*.

**Table 4:** Species, GenBank accession number, geographical locality, host, and references of Aspidoderidae GenBank sequences used in this study

Species	Accession number	Geographical locality	Host	References
*Aspidodera raillieti*	OL676808-OL676982	Brazil	*D. aurita* or *D. albiventris* or *P. quica*	Present study
*Aspidodera scoleciformis*	KC470136	Paraguay	*Euphractus sexcinctus*	[2]
*Aspidodera sogandaresi*	KC470131	Mexico	*Dasypus novemcinctus*	[2]
*Aspidodera kinsellai*	KC470134	Mexico	*D. novemcinctus*	[2]
*A. kinsellai*	KC470133	Mexico	*D. novemcinctus*	[2]
*A. kinsellai*	KC470132	Costa Rica	*D. novemcinctus*	[2]
*Lauroia trinidadensis*	KC470135	Mexico	*D. novemcinctus*	[2]
*Nematomystes rodentophilus*	KC470130	Argentina	*Oxymycterus paramensis*	[2]
*N. scapteromi*	KC470129	Argentina	*Scapteromys aquaticus*	[2]

At each dataset, we aligned the MT-CO1 sequences using the TranslatorX web server [38], employing amino acid translations to align protein-coding nucleotide sequences, using the MUSCLE algorithm [39]. The resulting alignments were manually trimmed of poorly aligned regions using the Mesquite software package, version 3.61 [40]. The presence of pseudogenes was checked using a phylogenetic method (PhyPA) based on pairwise alignment [41]. Substitution saturation in the matrices was assessed via the Xia test [42, 43]. Both tests were conducted using the DAMBE software package, version 6.4.79 [44].

Phylogenetic analyses under maximum likelihood (ML) as optimality criteria were generated using the PhyML, version 3.0 online web server [45]. Evolutionary model selection was implemented with SMS (Smart Model Selection) [46] in PhyML using the Akaike information criterion (AIC). Node support was assessed by the approximate likelihood-ratio test (aLRT) and by non-parametric bootstrap percentages (ML-BP) after 1000 replicates.

Bayesian inference (BI) analyses were performed using MrBayes, version 3.2.6 [47], executed on XSEDE through the CIPRES Science Gateway [48]. Independent GTR + I + G (general time-reversible nucleotide substitution model, with a proportion of invariable sites and gamma distribution of rates among sites) models were used for each codon position, with unlinking of base frequencies and parameters. Markov chain Monte Carlo (MCMC) sampling was performed for 10,000,000 generations with four simultaneous chains in two runs. Node support was assessed by Bayesian posterior probabilities (BPP), calculated from trees sampled every 100 generations, after removing the first 25% ‘burn-in’ generations. Sampling adequacy was assessed using the program Tracer, version 1.6 [49], to calculate the effective sample sizes (ESSs) of the parameters. Values above 200 effectively independent samples were considered robust.

Haplotype networks were inferred using the program PopART, version 1.7 [50], under the median-joining method [51]. We used DNAsp, version 5.10.1 [52], to organize *A. raillieti* sequences into groups according to (1) the clades recovered in the ML and BI phylogenetic trees, (2) the host species, and (3) the geographical localities. Additionally, using DNAsp, the genetic diversity of each group was calculated by the numbers of haplotypes (H), polymorphic sites (S), haplotype diversity (Hd), and nucleotide diversity (π).

### Population genetic analyses

Analysis of molecular variance (AMOVA) [53] and fixation index (*F*_st_) [54] were calculated using the Arlequin software package, version 3.5.2.2 [55]. We used AMOVA to analyse genetic variability between and within previously defined groups and *F*_st_ to measure levels of genetic differentiation between groups. We also used the program Arlequin to assess deviation from neutrality using Tajima’s D [56] and Fu’s Fs [57] tests for each of the previously determined groups.

The Mantel test [58] was used to verify the correlation between genetic distances and geographical distances of *A. raillieti* specimens, as well as the correlation between genetic distances and elevation differences from all geographical localities studied. The genetic distance matrix was calculated in the package ‘ape’ [59] using the evolutionary model selected in the automated model selection feature of the program PAUP*, version 4.0a167 [60], under AICc. The geographical distance matrix was built from the geographical coordinates of the studied localities using the package ‘fields’ [61]. The elevation difference matrix was generated from the elevation of each locality using the package ‘vegan’ [62]. All procedures of the Mantel test were computed within the R software environment, version 4.0.2 [35]. All statistical analyses were performed at the 5% significance level.

## Results

### Discriminant analysis of principal components

The number of PCs retained in the DAPC varied according to the investigated group. For the comparison between localities, 11 and 14 PCs were retained for female and male *A. raillieti*, respectively. For the comparison between host species, eight and 14 PCs were retained for female and male *A. raillieti*, respectively. The proportion of male specimens correctly classified to their original group was approximately 83% for locality and 75% for host species. The proportion of female specimens correctly classified was 75% for localities and 67% for host species.

**Fig. 2:**
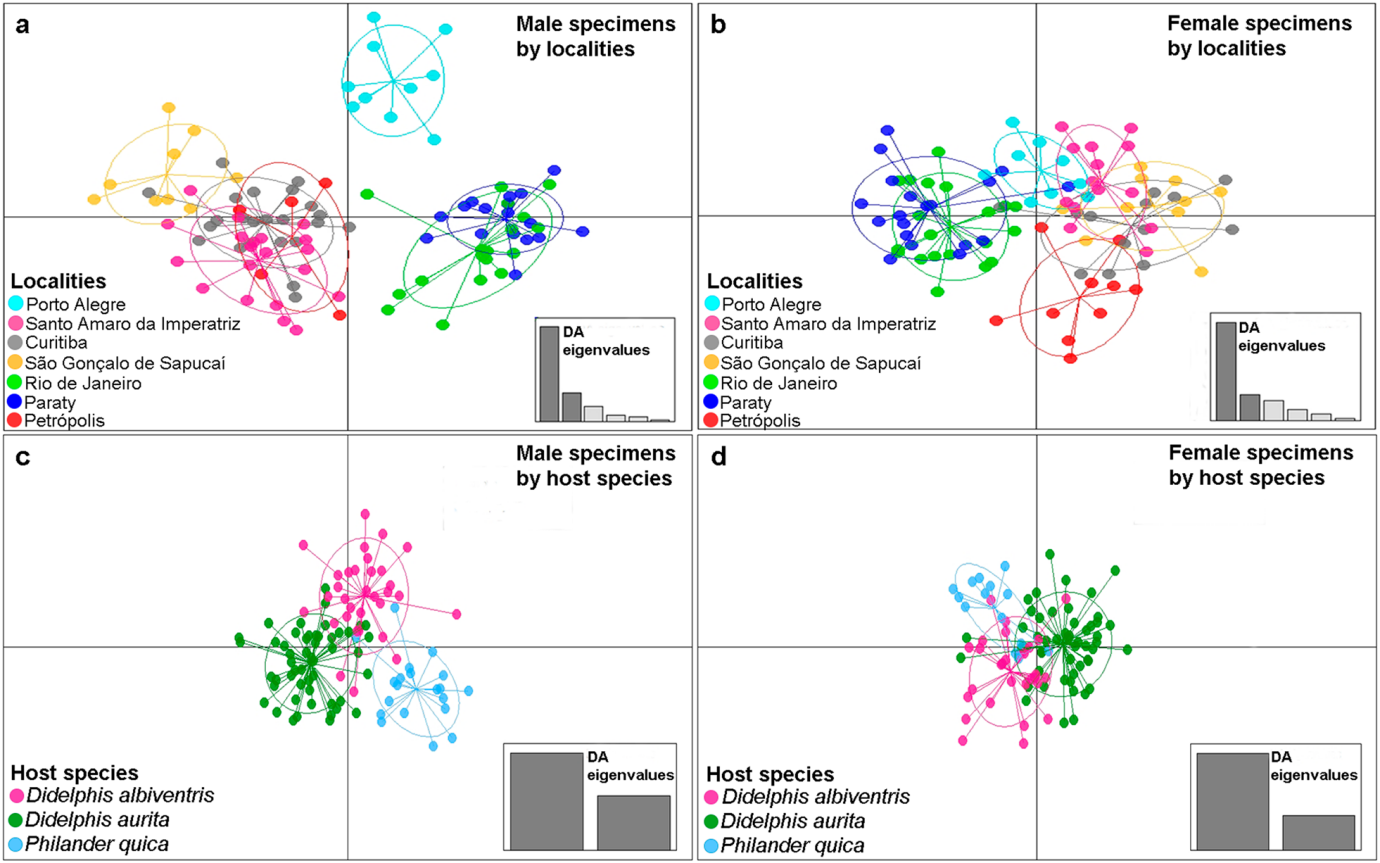
**a**, **c** Population clusters for males and females. **b**, **d** *Aspidodera raillieti* specimens based on DAPC along with discriminant analysis (DA) eigenvalues, showing morphometric variations between **a**, **b** localities and **c**, **d** host species

We observed greater morphometric variation in parasites among localities than among host species (Fig. 2). Furthermore, this result was more evident among male *A. raillieti*, revealing three clusters, each cluster formed by specimens with high morphometric proximity. One of these clusters was formed by the locality of Porto Alegre, Rio Grande do Sul (POA-RS); the second was formed by the localities of São Gonçalo do Sapucaí, Minas Gerais (SGS-MG), Santo Amaro da Imperatriz, Santa Catarina (SAI-SC), Curitiba, Paraná (CUR-PR), and Petrópolis, Rio de Janeiro (PET-RJ); and the third was formed by Rio de Janeiro, Rio de Janeiro (RIO-RJ) and Paraty, Rio de Janeiro (PTY-RJ) (Fig. 2a).

**Fig. 3:**
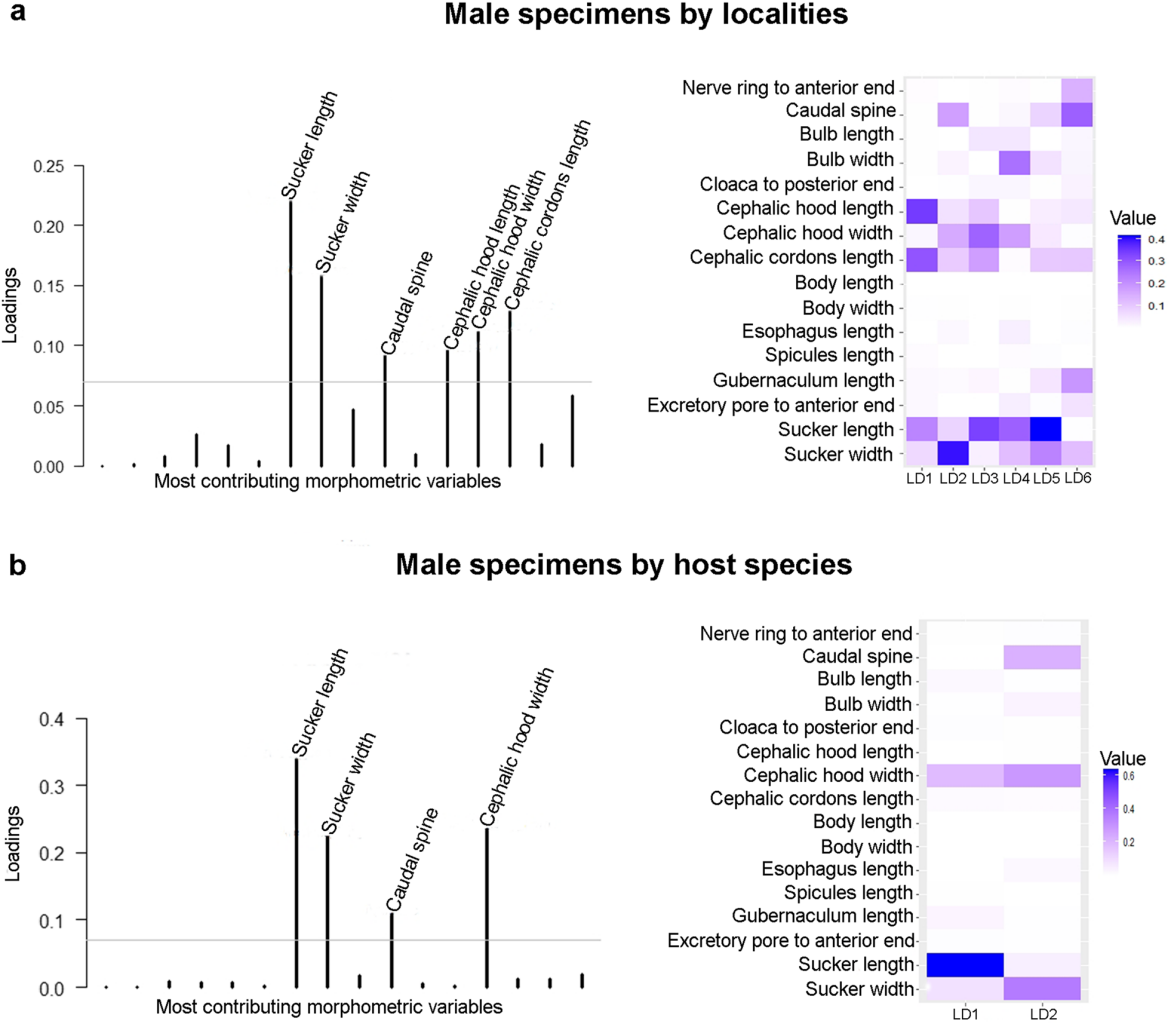
Morphometric variable contributions to *A. raillieti* male specimens. **a** Variables that best discriminated locality groups. **b** Variables that best discriminated host species groups

**Fig. 4:**
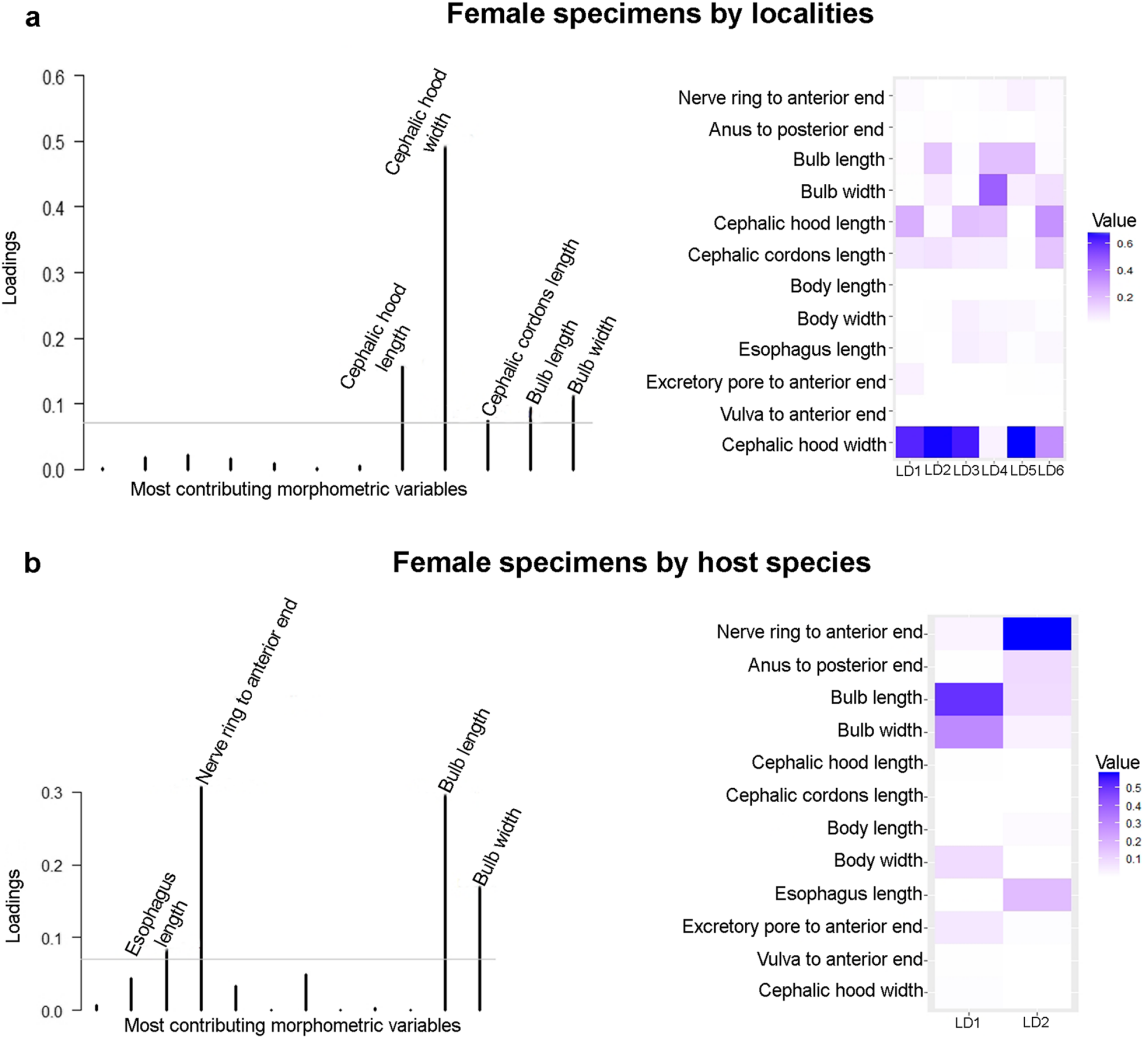
Morphometric variable contributions to *A. raillieti* female specimens. **a** Variables that best discriminated locality groups. **b** Variables that best discriminated host species groups

Among the variables that best discriminated among locality groups for males were length and width of the sucker, length and width of the hood, length of the cordon, and length of the caudal spine (Fig. 3a). For females, the variables were cephalic hood length, cephalic hood width, cephalic cordon length, bulb length, and bulb width (Fig. 4a). The variables that best discriminated parasites between host species for males were length and width of the sucker, width of the hood, and length of the caudal spine (Fig. 3b). For females, these variables included the distance of the nerve ring to the anterior end, length and width of the bulb, and length of the oesophagus (Fig. 4b).

### Phylogenetic analyses and genetic diversity

We successfully sequenced adult worms recovered from 17 *D. aurita*, nine *D. albiventris*, and four *P. quica* hosts collected in eight different localities. We obtained 489 bp MT-CO1 gene consensus sequences from 175 *A. raillieti* specimens. The first matrix, used for phylogenetic inferences, resulted in 183 taxa and 480 sites. The second matrix, used for phylogeographic analyses, included only our 175 sequences of *A. raillieti* with 489 sites. No long branch was observed in the PhyPA method, indicating the absence of pseudogenes. Xia’s test provided no evidence for substitution saturation in any data matrix.

The ML best-fit model chosen by SMS in PhyML under AIC was GTR + G, with four substitution rate categories and gamma shape parameter α = 0.095, resulting in a tree with lnL = −3030.323236 score. The BI sampling, after 25% ‘burn-in’, resulted in a mean estimated marginal likelihood of –3129.3479 (standard deviation = 18.2293; median = −3128.849). The ESSs were robust for all parameters.

**Fig. 5:**
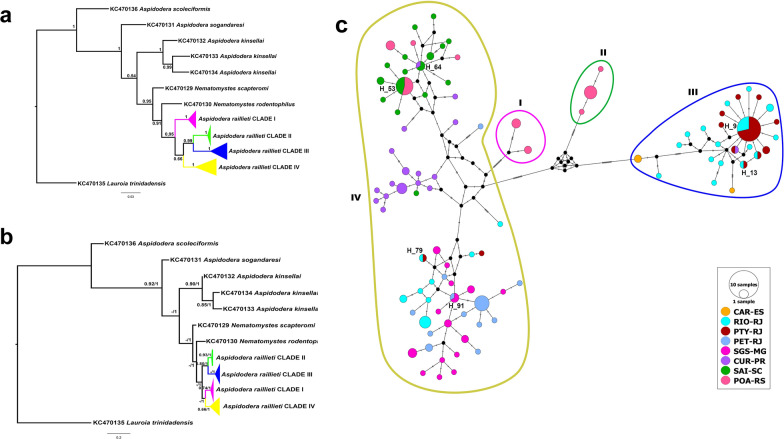
**a**, **b** Phylogenetic trees of partial MT-CO1 sequences of *A. raillieti* from this study and of aspidoderid species from GenBank. The *Lauroia trinidadensis* sequence was added as an out-group. Clades of *A. raillieti* are collapsed: clade I pink, clade II green, clade III blue, clade IV yellow. **a** Bayesian inference topology. Node values are BPP. **b** Maximum likelihood topology. Node values are ML-BP and aLRT > 0.50 support. **c** Median-joining network of partial MT-CO1 sequence haplotypes of *A. raillieti* from this study. Circle sizes are proportional to haplotype frequencies, and colours represent the localities where each haplotype occurs. Lines circling groups of haplotypes delimit the four clades recovered in our phylogenetic analyses. Circles identified by numbers in the haplotype network represent haplotypes shared between localities. Each hatch mark along the lines connecting haplotypes in the median-joining networks represents one mutation

Tree topologies from ML and BI analyses were similar, with some variations in nodes and support values. *Aspidodera raillieti* specimens formed a monophyletic group in both phylogenies (BP-ML = 0.42, aLRT = 1.00, BPP = 0.95). Within *A. raillieti*, four main monophyletic groups were recovered and identified as clades I, II, III, and IV (Fig. 5a, b).

**Fig. 6:**
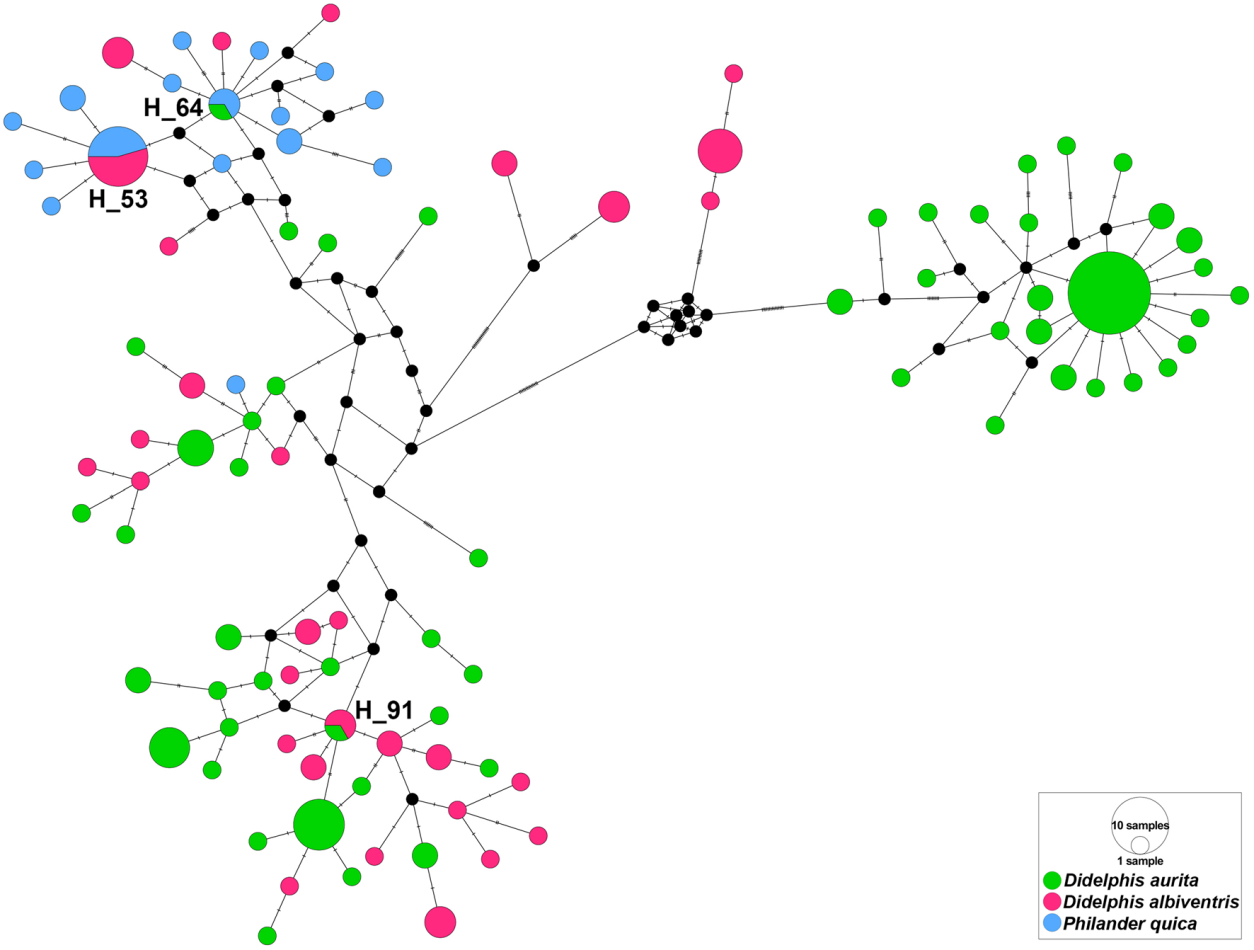
Geographical distribution of the clades identified in this study and the number of haplotypes for each clade by studied localities

Clade I (BP-ML = 0.74, aLRT = 1.00, BPP = 1.00) comprised *A. raillieti* parasites of *D. albiventris* from POA-RS (Additional file 2: Fig. S1). Clade II (BP-ML = 0.93, aLRT = 1.00, BPP = 1.00) comprised parasites of *D. albiventris* from POA-RS (Additional file 3: Fig. S2). Clade III (BP-ML < 0.5, aLRT = 1.00, BPP = 1.00) comprised parasites of *D. aurita* from CAR-ES, RIO-RJ, PTY-RJ, and CUR-PR (Additional file 4: Fig. S3). Finally, clade IV (BP-ML = 0.66, aLRT = 1.00, BPP = 1.00) comprised parasites of *D. aurita* from RIO-RJ, PTY-RJ, PET-RJ, and CUR-PR; parasites of *D. albiventris* from SGS-MG, CUR-PR, and POA-RS; and parasites of *P. quica* from SAI-SC (Additional file 5: Fig. S4, Fig. 6).

In the BI topology (Fig. 5a), clade I was recovered as sister to clades II, III, and IV (BPP = 0.95), whereas clades II and III formed a monophyletic group (BPP = 0.99), sister to clade IV (BPP = 0.66). In the ML topology (Fig. 5b), clades II and III formed a monophyletic group (aLRT = 1.00, BP-ML = 0.80), sister to a monophyletic group formed by clades I and IV (aLRT = 1.00, BP-ML > 0.5).

### Haplotype networks

Among our 175 partial MT-CO1 gene sequences (489 bp) of *A. raillieti*, we identified 99 haplotypes with 114 polymorphic sites. These 99 haplotypes were grouped into four haplogroups, corresponding to the clades recovered in the phylogenies, separated by genetic distances of 20 to 30 mutational steps (Fig. 5c). The molecular diversity indices of groups were separated by the clades recovered in phylogenies, host species, and localities. All groups studied (clades, host species, and localities) had prominent levels of haplotype diversity but low levels of nucleotide diversity (Table 5).

**Table 5:** Genetic diversity indices for *A. raillieti* sequence groups divided by clades, host species, and localities

	*N*	*H*	*S*	Hd	Π
Haplogroup					
Clade I	5	2	7	0.6	0.00859
Clade II	8	3	3	0.464	0.00153
Clade III	51	25	40	0.831	0.00737
Clade IV	111	69	70	0.98	0.02121
Host					
*D. albiventris*	53	30	78	0.967	0.03905
*D. aurita*	99	56	89	0.946	0.04680
*P. quica*	23	16	25	0.949	0.00815
Locality					
CAR-ES	3	2	15	0.667	0.02045
RIO-RJ	35	24	60	0.955	0.04252
PTY-RJ	27	12	47	0.698	0.01295
PET-RJ	20	12	29	0.847	0.00964
SGS-MG	22	15	17	0.965	0.00839
CUR-PR	23	19	60	0.972	0.01851
SAI-SC	23	16	25	0.949	0.00815
POA-RS	22	7	56	0.84	0.04765

*N*: sample size; H: number of haplotypes; S: number of polymorphic sites; Hd: haplotype diversity; π: nucleotide diversity

The localities RIO-RJ and PTY-RJ included clades III and IV haplotypes. The locality POA-RS included clades I, II, and IV haplotypes. The locality CUR-PR had only one clade III haplotype, while all others had clade IV haplotypes. Haplotypes from the localities CAR-ES (clade III), PET-RJ (clade IV), SGS-MG (clade IV) and SAI-SC (clade IV) clustered in only one haplogroup per locality (Fig. 5c). Some haplotypes were shared between localities. Haplotypes 9, 13, 21, and 79 were shared between the RIO-RJ and PTY-RJ localities. Haplotype 91 was shared between PET-RJ and SGS-MG. Haplotype 16 was shared between PTY-RJ and CUR-PR. Haplotype 64 was shared between CUR-PR and SAI-SC. Finally, haplotype 53 was shared between POA-RS and SAI-SC (Fig. 5c).

**Fig. 7:**
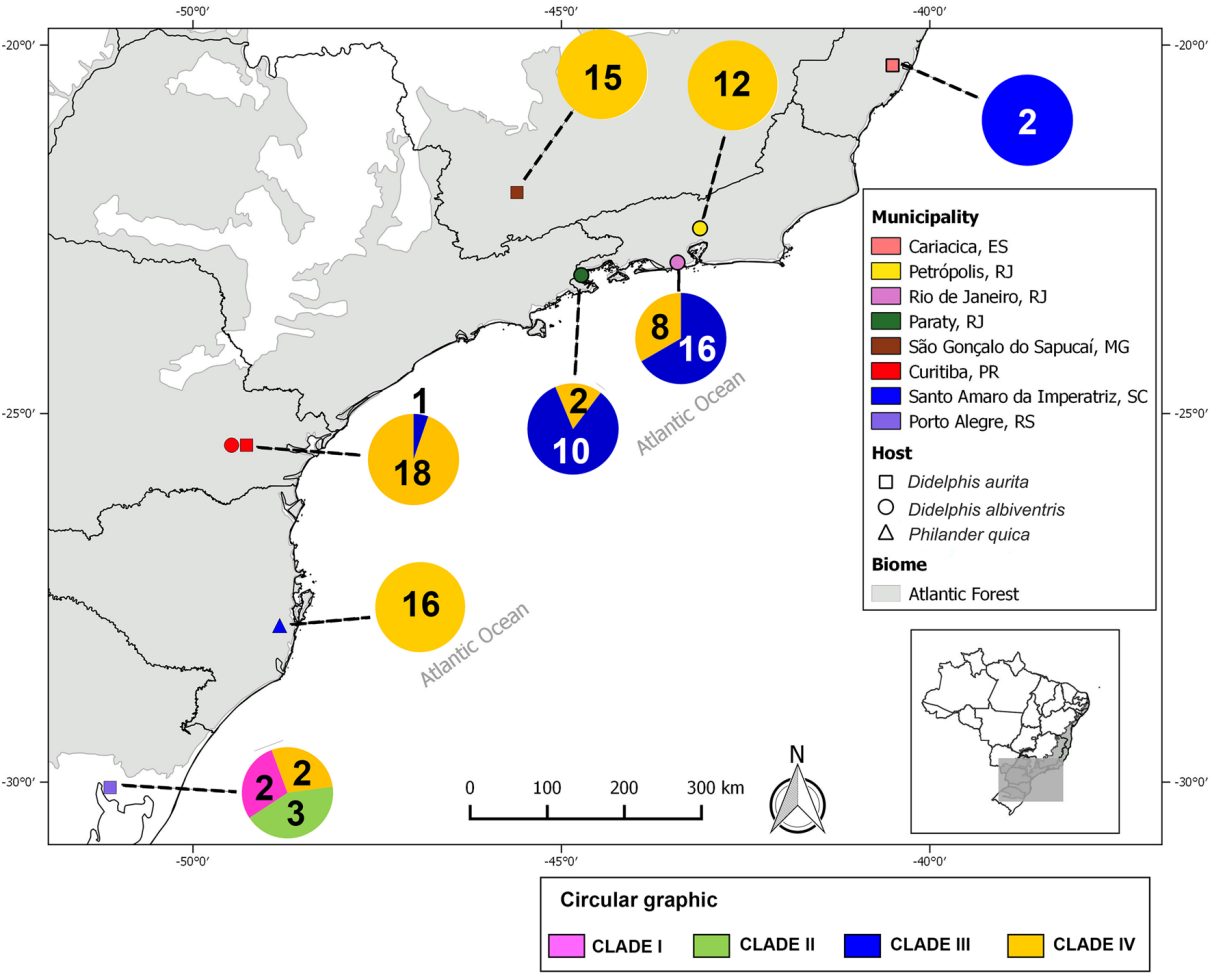
Median-joining network for partial MT-CO1 sequence haplotypes of 175 *A. raillieti* sequences from the present study. Circle sizes are proportional to haplotype frequencies, and the colours represent the hosts (*D. aurita*, *D. albiventris*, and *P. quica*) in which each haplotype occurs. Each hatch mark along the lines connecting haplotypes represents one mutation. Only haplotypes shared between hosts have their identifications represented in the haplotype network

We also observed haplotype sharing between host species. Haplotype 91 was shared between *D. aurita* (PET-RJ) and *D. albiventris* (SGS-MG). Haplotype 64 was shared between *D. aurita* (CUR-PR) and *P. quica* (SAI-SC). Haplotype 53 was shared between *D. albiventris* (POA-RS) and *P. quica* (SAI-SC) (Fig. 7).

### Population structure

The AMOVA result for clade groups revealed higher variation among clades, which represented 80.64% of the total variation, while the variation within clades represented 19.36%. The genetic variation for the host species groups revealed less variation among host species, which represented 28.50% of the variation, while the variation within host species represented 71.50%. The genetic variation for locality groups revealed higher variation among localities, which represented 57.52% of the variation, while the variation within localities represented 42.48% (Table 6).

**Table 6:** Analysis of molecular variance (AMOVA) of *A. raillieti* among and within clades recovered in phylogenetic inferences, host species, and geographical localities

Source of variation	df	Sum of squares	Variance components	Percentage variation	*P-*value
Clades					
Among clades	3	1509.248	16.78005Va	80.64	0.00000
Within clades	171	688.755	4.02780Vb	19.36	
Total	174	2198.002	20.80786		
Hosts					
Among hosts	2	429.975	4.09745Va	28.50	0.00000
Within hosts	172	1768.027	10.27923Vb	71.50	
T otal	174	2198.002	14.37667		
Geographic locality					
Among localities	7	1224.455	7.89519Va	57.52	0.00000
Within localities	167	973.548	5.82963Vb	42.48	
Total	174	2198.002	13.72482		

*P* < 0.05, df: degrees of freedom

**Fig. 8:**
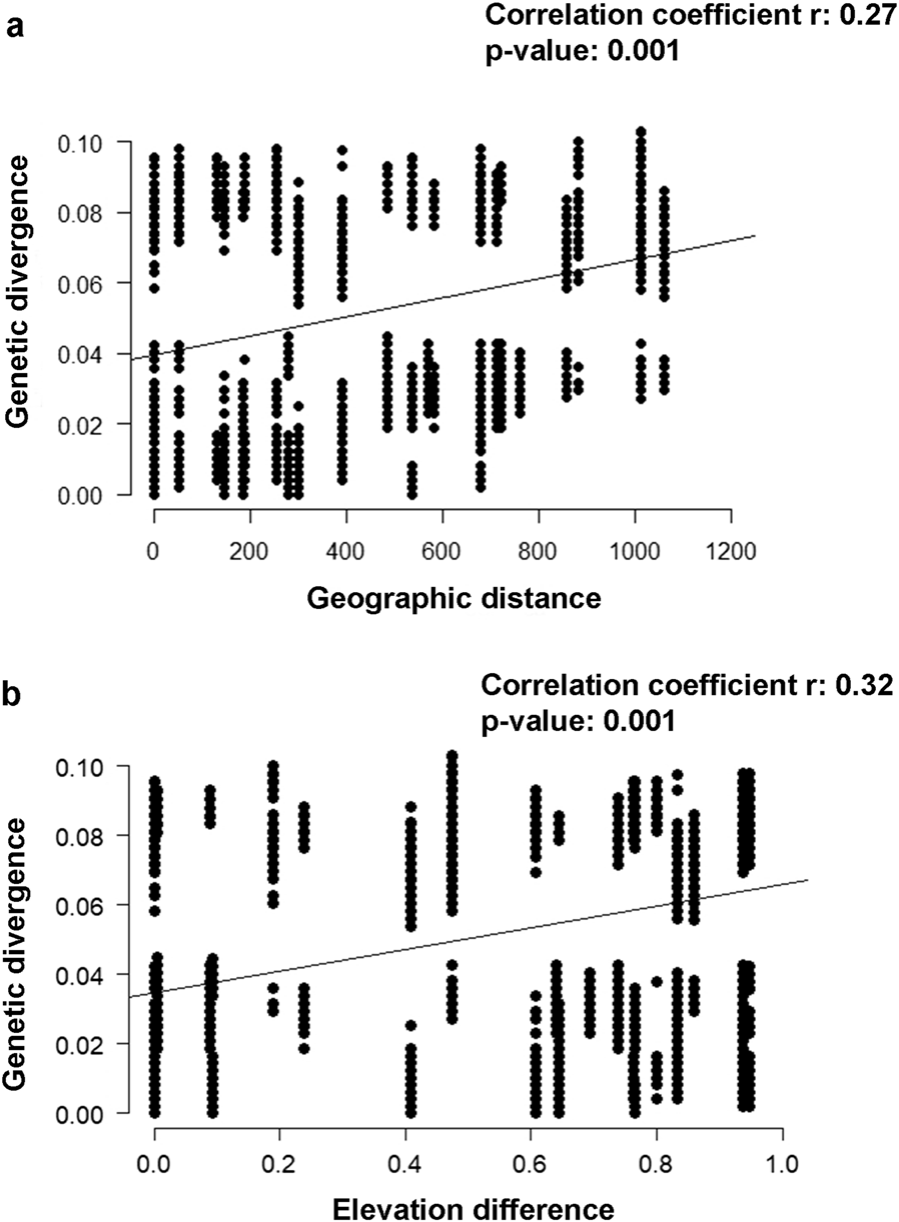
**a** Mantel test of correlation between genetic divergences and geographical distances; **b** Mantel test of correlation between genetic divergences and elevation differences

Considering the fixation index, all *F*_st_ values revealed significant genetic differences between clades (*P* < 0.05), host species (*P* < 0.01) (Table 7), and locality groups (*P* < 0.05). No difference was observed between RIO-RJ and CAR-ES or between PTY-RJ and CAR-ES (Table 8). The Mantel tests executed using the Kimura two-parameter nucleotide substitution model, calculated using PAUP, indicated a significant and positive correlation between genetic distance and geographical distance (*N* = 175, *r* = 0.27, *P* = 0.001) (Fig. 8a) and a significant and positive correlation between genetic distance and elevation difference (*N* = 175, *r* = 0.32, *P* = 0.001) (Fig. 8b).

**Table 7:** Pairwise *F*_st_ values of *A. raillieti* sequences between clades and host species groups

	\Clade I	Clade II	Clade III	Clade IV
Clade I				
Clade II	0.47962*			
Clade III	0.24468*	0.29764*		
Clade IV	0.15199*	0.20686*	0.09133*	

Host species	*D. albiventris*	*D. aurita*	*P. quica*
*D. albiventris*			
*D. aurita*	0.04366**		
*P. quica*	0.01793**	0.05206**	

*Significant results (*P* < 0.05), **significant (*P* < 0.01)

**Table 8:** Pairwise *F*_st_ values of *A. raillieti* sequences between geographical locality groups

	CAR-ES	RIO-RJ	PTY-RJ	PET-RJ	SGS-MG	CUR-PR	SAI-SC
CAR-ES							
RIO-RJ	0.13322						
PTY-RJ	0.31018	0.08083*					
PET-RJ	0.20659*	0.09648*	0.23075*				
SGS-MG	0.12899*	0.04014*	0.17171*	0.08898*			
CUR-PR	0.12426*	0.03672*	0.16614*	0.08936*	0.03114*		
SAI-SC	0.13935*	0.04831*	0.17924*	0.10133*	0.04303*	0.03588*	
POA-RS	0.21114*	0.10052*	0.23331*	0.15645*	0.09740*	0.09365*	0.04924*

*Significant results (*P* < 0.05)

### Historical demography

In Tajima’s D and Fu’s Fs neutrality tests, calculated for the clades, D values (−2.02) were significant only for clade III (*P* = 0.005), while Fs values were significant for both clade III (−15.09) and clade IV (−24.39) (*P* = 0.000). For the host species groups, the D and Fs values were significant only for the group of *A. raillieti* parasites of *P. quica* (−1.545, *P* = 0.05 and −8.333, *P* = 0.0004, respectively). For the locality groups, D values were significant only for the localities PTY-RJ (−1.819, *P* = 0.024), PET-RJ (−1.654, *P* = 0.027) and CUR-PR (−1.746, *P* = 0.015), whereas Fs values were significant for the localities CUR-PR (−6.942, *P* = 0.007), SGS-MG (−6.931, *P* = 0.000), and SAI-SC (−8.333, *P* = 0.003).

## Discussion

### Morphometric comparison between *A. raillieti* specimens from different localities and hosts.

The DAPC for *A. raillieti* specimen groups associated with different hosts (*D. albiventris*, *D*. *aurita*, and *P. quica*) indicated morphometric differentiation for both females and males, however, with partial overlapping of some specimens from different host species.

Among the variables that best discriminated *A. raillieti* specimens associated with host species and localities, our results identified the cephalic hood width for male and female specimens, the sucker length and width for females, and the bulb length and width for females. This morphometric variability may be a consequence of adaptations to environmental conditions, as observed in the trematode *Echinostoma paraensei* Lie & Basch, 1967 [63].

When analysing DAPC morphometric differentiation between *A. raillieti* specimen groups associated with different localities, female nematodes had greater overlap between localities, while males were differentiated into three groups. The first group was composed of male specimens from the localities RIO-RJ and PTY-RJ, the second was composed of specimens from POA-RS, and the third was composed of specimens from PET-RJ, SGS-MG, CUR-PR, and SAI-SC.

Our findings indicated the influence of host species, as well as locality, on the morphometry of *A. raillieti*, thus suggesting phenotypic plasticity regarding host attributes and spatial variables [64].

### Population structure of *A. raillieti*

Population structure can be influenced by different evolutionary forces. Among them, gene flow is considered of fundamental importance, as it allows the exchange of genetic information between populations, homogenizing the variation among them [65]. Population genetic studies of parasitic helminths have shown that gene exchange between populations is strongly influenced by the movement of their vertebrate [17, 22] and invertebrate hosts [66]. *Aspidodera raillieti* is a parasite with a monoxenous life cycle [10], depending on its mammalian hosts for dispersion. Thus, its population structure is expected to be dependent on the movement and encounter of mammalian hosts.

Our phylogenetic trees and networks recovered four clades of *A. raillieti*. These results provide evidence that these lineages experienced past events that contributed to the genetic divergence observed between clades, since the genetic structuring observed in these helminths was not related to geographical distance, as evidenced by the presence of divergent clades in the same locality.

The genetic structure observed using AMOVA indicated a greater genetic variation among clades (interpopulation) than within clades (intrapopulation). The highly significant *F*_st_ values, indicating high genetic differentiation between clades [67], concurred with the AMOVA results, also indicating limited gene flow between the four clades. However, analysing other genetic markers from independent loci would be necessary to deliver a clearer picture of the evolutionary history of *A. raillieti*.

No geographical structuring was observed, since the AMOVA results had similar percentages of genetic variation among and within localities (57.52% and 42.48%, respectively). This was congruent with *F*_st_, with high values both between geographically distant localities and between closer localities. Nevertheless, the Mantel test showed a significant and positive correlation between genetic and geographical distances, indicating isolation by distance (IBD). The disagreement between the population structure analyses and the Mantel test may be a consequence of the co-occurrence of haplotypes from different clades in the same locality, possibly due to historical processes. The phylogeographic patterns of species can be affected by different factors, such as dispersal or vicariant events, which can promote differentiation between populations [65]. In addition, it has been postulated that the current distributions of several lineages of mammals, birds, and amphibians in South America originated from several mechanisms, such as Quaternary climatic oscillations and Tertiary orogenic events [68, 69]. However, to identify which event led to the divergence between the lineages recovered in our analyses, a well-calibrated molecular clock would be necessary to estimate the divergence times between them compared to known events [65].

The high genetic diversity observed in *A. raillieti* has also been identified in populations of the nematode *Heligmosomoides polygyrus* (Dujardin, 1845), a parasite of the forest rodent *Apodemus sylvaticus* (Linnaeus, 1758). To understand the phylogeographic pattern of *H. polygyrus* from different localities in Europe, Nieberding et al. [70] studied the genetic structure of the populations of this monoxenous nematode. The authors found a high number of haplotypes for the MT-CYB gene, totalling 126 haplotypes from 136 sequenced specimens. Five main groups were observed, both in phylogenetic reconstructions and haplotype networks, which showed a high degree of genetic divergence, being separated by a genetic distance of 18 to 35 mutational steps, as observed between *A. raillieti* clades (20 to 30 mutational steps), with some haplotypes co-occurring in some localities.

Similarly, the infective free-living stages of *H. polygyrus* and *A. raillieti* have no specialized structures for dispersal; thus, gene flow between populations depends mainly on host movements and social behaviour. However, *H. polygurus* is a host specialist and has a wide distribution congruent with its host, partially reflecting the phylogeographic history of its host [70, 71]. In contrast, *A. raillieti* is a host-generalist parasite that is able to infect several marsupials and one rodent species, which makes its phylogeographic patterns potentially more complex, requiring studies with broader geographical ranges and other host species to better understand its genetic population structure.

Analysing *A. raillieti* by host species, the phylogenetic and phylogeographic results showed no evidence of population specificity for host species, unlike the morphometric analyses, since nematode haplotypes from different marsupial species were in the same clade. Additionally, some haplotypes were shared between different host species. Corroborating this pattern, no structuring was observed in *A. raillieti* associated with host species, as AMOVA indicated low genetic variation between the specimens recovered from each host species (interhost). The significant *F*_st_ values between host species indicated moderate genetic differentiation. Moreover, this genetic differentiation may result partially from geographical distances among localities and from divergent clades.

Some ecological characteristics of the host species studied promote unfavourable conditions for the emergence of parasite population genetic structuring patterns. *Didelphis aurita* and *D. albiventris* are omnivorous frugivores, while *P. quica* is an omnivorous insectivore, and all have overlapping diets [72]. They also have the same locomotor habits, as both are scansorial [73, 74]. However, *D. albiventris* is a habitat generalist, while the other two species mostly occur in forested regions [75, 76]. In addition, both *D. aurita* and *D. albiventris* occur in abundance in degraded areas [77], unlike *P. quica* [29]. Although Cáceres et al. [23] have shown that in areas of sympatry, *D. aurita* and *D. albiventris* explore different niches, this barrier may not have been sufficient to prevent gene flow between populations of *A. raillieti* in these hosts. We also observed a significant correlation between genetic divergence and elevation differences. The geographical distribution of *D. albiventris* is larger than that of *D. aurita*, as the former is a more habitat generalist and has greater climatic tolerance than the latter, which is also reflected in the elevation.

Moreover, we expected to find greater genetic differentiation between parasites recovered from *P. quica* and *D. albiventris* than between parasites recovered from *P. quica* and *D. aurita*, as the distribution of *P. quica* overlaps that of *D. aurita* but not completely that of *D. albiventris* [12, 29]. As *D. aurita* and *P. quica* have niche overlap, compete [27, 28], and may have intraguild predation [30], all these characteristics may favour parasite gene flow between these host populations. However, less genetic differentiation was found between *A. raillieti* specimens recovered from *P. quica* and *D. albiventris* than between *A. raillieti* specimens recovered from *P. quica* and *D. aurita*. This may be because *A. raillieti* samples of *P. quica* were collected from a single locality, closer to *D. albiventris* than to *D. aurita* localities.

As observed in *A. raillieti* populations, the nematode *Trichostrongylus axei* (Cobbold, 1879), a host-generalist parasite that infects multiple sympatric wild ungulates, showed no evidence of genetic structure associated with host species [20]. The authors proposed that *T. axei* populations would be structured due to the degree of spatial niche partitioning between hosts.

López-Caballero et al. [78] performed a study on the genetic divergence of populations of the acanthocephalan *Oligacanthorhynchus microcephalus* (Rudolphi, 1819) parasitizing three definitive hosts of the tribes Didelphini, *Didelphis marsupialis*, *D. virginiana*, and *Philander opossum* from different localities in Mexico. Phylogenetic analyses demonstrated a similar pattern to that found for *A. raillieti*, in which the specimens of *O. microcephalus* were grouped into three main clades, which were not correlated either with definitive host species or with geographical distributions. The lack of population structuring was attributed to several aspects, including host natural histories, dispersal abilities, sympatries, overlapping diets, and the fact that the arthropod intermediate hosts of *O. microcephalus* are distributed throughout the entire geographical range of this parasite.

### Demographic history of *Aspidodera raillieti*

Climate changes, such as Pleistocene glaciations, promoted the retraction of tropical forests, forming refuges and the subsequent expansion of these forests due to climate amelioration. Populations from refuge areas that undergo postglacial demographic expansion have consequent genetic signatures [79, 80]. These demographic fluctuations can be detected by some analyses, such as the neutrality tests used in this study [56, 57]. Significant negative D or Fs values in neutrality tests suggest a population undergoing purifying selection or expansion, characterized by an excess of rare alleles [67].

In our neutrality tests, parasitic specimens from the PTY-RJ, PET-RJ, CUR-PR, SGS-MG, and SAI-SC localities had significant negative values, some of which were negative for Tajima’s D or Fu’s Fs. This expansion signature was congruent with the genetic diversity indices, showing high haplotype diversity and low nucleotide diversity for the MT-CO1 gene for all groups studied (clades, localities, and hosts). These results also showed that although there were many haplotypes, they differed from each other by only a few nucleotide substitutions. This pattern is consistent with a rapidly expanding population from a small effective population size [81].

Since *A. raillieti* has a Neotropical and partially Nearctic distribution, future studies should include specimens from other biomes, encompassing its entire distribution range, to better understand the evolutionary history of this parasite. It would also be necessary to include nuclear genetic markers from independent loci to verify whether the phylogeographic pattern observed for the MT-CO1 gene is corroborated. Additionally, the inclusion of a time scale to estimate divergence times between clades would make it possible to verify congruence between cladogenesis and palaeogeographical and climatic events [71, 82].

## Conclusion

Based on our results, we concluded that the genetic structure of *A. raillieti* populations in the South and Southeast Atlantic Forest was likely associated with historical events, such as past climate changes, and not with the host species *D. aurita*, *D. albiventris*, and *P. quica* or with the current geographical distribution of this parasitic nematode. We also observed greater morphometric variation than molecular structuring associated with host species and localities, suggesting phenotypic plasticity related to host functional traits, as well as to spatial variables.

## Supplementary Information

**Additional file 1: Table S1.** Municipality and state of origin, hosts, and GenBank accession number of MT-CO1 gene sequences of 175 *A. raillieti* specimens from this study.

**Additional file 2: Figure S1.** Bayesian phylogenetic trees of partial MT-CO1 sequences of *A. raillieti* from this study and of aspidoderid species from GenBank. The sequence of *Lauroia trinidadensis* was added as an out-group. Clade I of *A. raillieti* is not collapsed.

**Additional file 3: Figure S2.** Bayesian phylogenetic trees of partial MT-CO1 sequences of *A. raillieti* from this study and of aspidoderid species from GenBank. The sequence of *Lauroia trinidadensis* was added as an out-group. Clade II of *A. raillieti* is not collapsed.

**Additional file 4: Figure S3.** Bayesian phylogenetic trees of partial MT-CO1 sequences of *A. raillieti* from this study and of aspidoderid species from GenBank. The sequence of *Lauroia trinidadensis* was added as an out-group. Clade III of *A. raillieti* is not collapsed.

**Additional file 5: Figure S4.** Bayesian phylogenetic trees of partial MT-CO1 sequences of *A. raillieti* from this study and of aspidoderid species from GenBank. The sequence of *Lauroia trinidadensis* was added as an out-group. Clade IV of *A. raillieti* is not collapsed.

## Data Availability

All the new *A. raillieti* MT-CO1 sequences obtained for this study were deposited in NCBI GenBank under accession numbers OL676808-OL676982.

## Abbreviations

m.a.s.l.: Meters above sea level
CAR-ES: Cariacica, Espírito Santo
PET-RJ: Petrópolis, Rio de Janeiro
RIO-RJ: Rio de Janeiro, Rio de Janeiro
PTY-RJ: Paraty, Rio de Janeiro
SGS-MG: São Gonçalo do Sapucaí, Minas Gerais
CUR-PR: Curitiba, Paraná
SAI-SC: Santo Amaro da Imperatriz, Santa Catarina
POA-RS: Porto Alegre, Rio Grande do Sul
NaCl 0.85%: Physiological saline solution
DAPC: Discriminant analysis of principal components
PCs: Principal components
MT-CO1: Mitochondrially encoded cytochrome c oxidase I
PCR: Polymerase chain reaction
PhyPA: Phylogenetic method
ML: Maximum likelihood
IB: Bayesian inference
SMS: Smart Model Selection
AIC: Akaike information criterion
aLRT: Approximate likelihood-ratio test
BP: Bootstrap percentage
GTR: General time-reversible nucleotide substitution model
I: proportion of invariable sites
G: gamma-distributed heterogeneity of substitution rates among sites
MCMC: Markov chain Monte Carlo
BPP: Bayesian posterior probabilities
H: Numbers of haplotypes
S: Polymorphic sites
Hd: Haplotype diversity
π: Nucleotide diversity
AMOVA: Analysis of molecular variance
*F*_st_: Fixation index
IBD: Isolation by distance
AICc: Corrected Akaike information criterion
ESS: Effective Sample Size
