# Supplementary material for: Population genetic structure and phenotypic diversity of Aspidodera raillieti (Nematoda: Heterakoidea), a parasite of Didelphini marsupials in Brazil’s South and Southeast Atlantic Forest
Source: Parasit Vectors. 2022 Jun 13;15:203. doi: 10.1186/s13071-022-05288-6 (PMC9195327; doi:10.1186/s13071-022-05288-6)
Supplement: Supplementary file 1 — Additional file 1: Table S1. Municipality and state of origin, hosts, and GenBank accession number of MT-CO1 gene sequences of 175 A. raillieti specimens from this study. [file 13071_2022_5288_MOESM1_ESM.docx]

**Table S1.** Municipality and state of origin, hosts and GenBank accession number of MT-CO1 gene sequences of 175 *A. raillieti* specimens from this study.

| Municipality state | Host | Accession number |
| --- | --- | --- |
| Cariacica, Rio de Janeiro | *Didelphis aurita* | OL676808 |
|  |  | OL676857 |
|  |  | OL676858 |
| Petrópolis, Rio de Janeiro | *Didelphis aurita* | OL676872 |
|  |  | OL676936 |
|  |  | OL676945 |
|  |  | OL676949 |
|  |  | OL676951 |
|  |  | OL676952 |
|  |  | OL676955 |
|  |  | OL676956 |
|  |  | OL676957 |
|  |  | OL676959 |
|  |  | OL676960 |
|  |  | OL676961 |
|  |  | OL676962 |
|  |  | OL676963 |
|  |  | OL676964 |
|  |  | OL676965 |
|  |  | OL676966 |
|  |  | OL676968 |
|  |  | OL676978 |
|  |  | OL676979 |
| Rio de Janeiro, Rio de Janeiro | *Didelphis aurita* | OL676809 |
|  |  | OL676810 |
|  |  | OL676811 |
|  |  | OL676812 |
|  |  | OL676813 |
|  |  | OL676814 |
|  |  | OL676815 |
|  |  | OL676817 |
|  |  | OL676818 |
|  |  | OL676820 |
|  |  | OL676823 |
|  |  | OL676824 |
|  |  | OL676838 |
|  |  | OL676839 |
|  |  | OL676840 |
|  |  | OL676845 |
|  |  | OL676847 |
|  |  | OL676849 |
|  |  | OL676850 |
|  |  | OL676854 |
|  |  | OL676855 |
|  |  | OL676856 |
|  |  | OL676927 |
|  |  | OL676929 |
|  |  | OL676930 |
|  |  | OL676931 |
|  |  | OL676932 |
|  |  | OL676933 |
|  |  | OL676934 |
|  |  | OL676937 |
|  |  | OL676938 |
|  |  | OL676939 |
|  |  | OL676940 |
|  |  | OL676941 |
|  |  | OL676947 |
| Paraty, Rio de Janeiro | *Didelphis aurita* | OL676816 |
|  |  | OL676819 |
|  |  | OL676821 |
|  |  | OL676822 |
|  |  | OL676826 |
|  |  | OL676827 |
|  |  | OL676828 |
|  |  | OL676829 |
|  |  | OL676830 |
|  |  | OL676831 |
|  |  | OL676832 |
|  |  | OL676833 |
|  |  | OL676834 |
|  |  | OL676835 |
|  |  | OL676836 |
|  |  | OL676837 |
|  |  | OL676841 |
|  |  | OL676842 |
|  |  | OL676843 |
|  |  | OL676844 |
|  |  | OL676846 |
|  |  | OL676848 |
|  |  | OL676851 |
|  |  | OL676852 |
|  |  | OL676853 |
|  |  | OL676928 |
|  |  | OL676946 |
| São Gonçalo do Sapucaí, Minas Gerais | *Didelphis albiventris* | OL676935 |
|  |  | OL676942 |
|  |  | OL676943 |
|  |  | OL676944 |
|  |  | OL676948 |
|  |  | OL676950 |
|  |  | OL676953 |
|  |  | OL676954 |
|  |  | OL676958 |
|  |  | OL676967 |
|  |  | OL676969 |
|  |  | OL676970 |
|  |  | OL676971 |
|  |  | OL676972 |
|  |  | OL676973 |
|  |  | OL676974 |
|  |  | OL676975 |
|  |  | OL676976 |
|  |  | OL676977 |
|  |  | OL676980 |
|  |  | OL676981 |
|  |  | OL676982 |
| Curitiba, Paraná | *Didelphis aurita* | OL676825 |
|  |  | OL676874 |
|  |  | OL676875 |
|  |  | OL676876 |
|  |  | OL676880 |
|  |  | OL676881 |
|  |  | OL676885 |
|  |  | OL676887 |
|  |  | OL676888 |
|  |  | OL676889 |
|  |  | OL676890 |
|  |  | OL676891 |
|  |  | OL676892 |
|  |  | OL676926 |
| Curitiba, Paraná | *Didelphis albiventris* | OL676873 |
|  |  | OL676877 |
|  |  | OL676878 |
|  |  | OL676882 |
|  |  | OL676883 |
|  |  | OL676884 |
|  |  | OL676886 |
|  |  | OL676913 |
|  |  | OL676921 |
|  |  | OL676879 |
| Santo Amaro da Imperatriz, Santa Catarina | *Philander quica* | OL676893 |
|  |  | OL676894 |
|  |  | OL676895 |
|  |  | OL676896 |
|  |  | OL676897 |
|  |  | OL676898 |
|  |  | OL676899 |
|  |  | OL676900 |
|  |  | OL676902 |
|  |  | OL676903 |
|  |  | OL676904 |
|  |  | OL676905 |
|  |  | OL676906 |
|  |  | OL676907 |
|  |  | OL676914 |
|  |  | OL676918 |
|  |  | OL676919 |
|  |  | OL676920 |
|  |  | OL676922 |
|  |  | OL676923 |
|  |  | OL676924 |
|  |  | OL676925 |
|  |  | OL676859 |
| Porto Alegre, Rio Grande do Sul | *Didelphis albiventris* | OL676860 |
|  |  | OL676861 |
|  |  | OL676862 |
|  |  | OL676863 |
|  |  | OL676864 |
|  |  | OL676865 |
|  |  | OL676866 |
|  |  | OL676867 |
|  |  | OL676868 |
|  |  | OL676869 |
|  |  | OL676870 |
|  |  | OL676871 |
|  |  | OL676901 |
|  |  | OL676908 |
|  |  | OL676909 |
|  |  | OL676910 |
|  |  | OL676911 |
|  |  | OL676912 |
|  |  | OL676915 |
|  |  | OL676916 |
|  |  | OL676917 |
|  |  |  |
