# Supplementary figures and images for: Population genetic structure and phenotypic diversity of Aspidodera raillieti (Nematoda: Heterakoidea), a parasite of Didelphini marsupials in Brazil’s South and Southeast Atlantic Forest
Source: Parasit Vectors. 2022 Jun 13;15:203. doi: 10.1186/s13071-022-05288-6 (PMC9195327; doi:10.1186/s13071-022-05288-6)

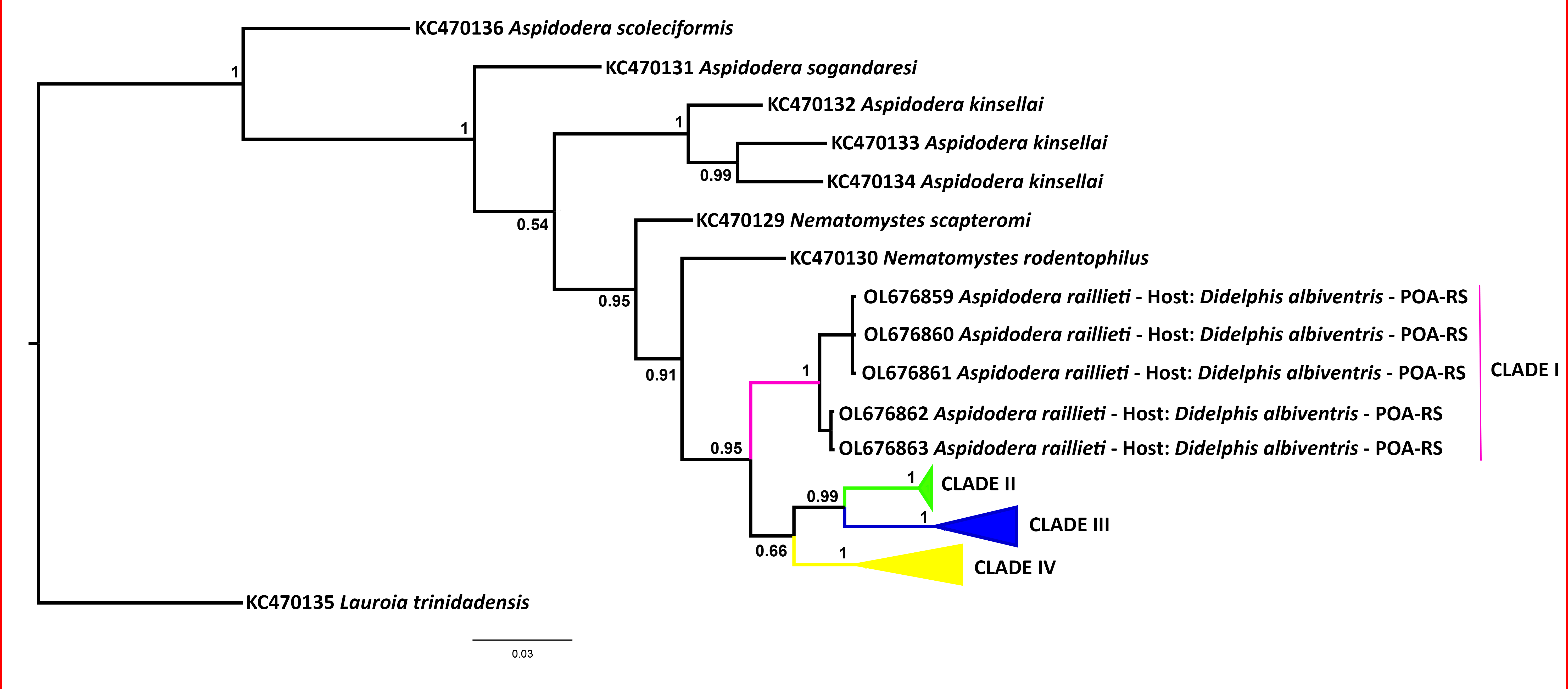

Supplement: Supplementary file 2 — Additional file 2: Figure S1. Bayesian phylogenetic trees of partial MT-CO1 sequences of A. raillieti from this study and of aspidoderid species from GenBank. The sequence of Lauroia trinidadensis was added as an out-group. Clade I of A. raillieti is not collapsed. [file 13071_2022_5288_MOESM2_ESM.tif]

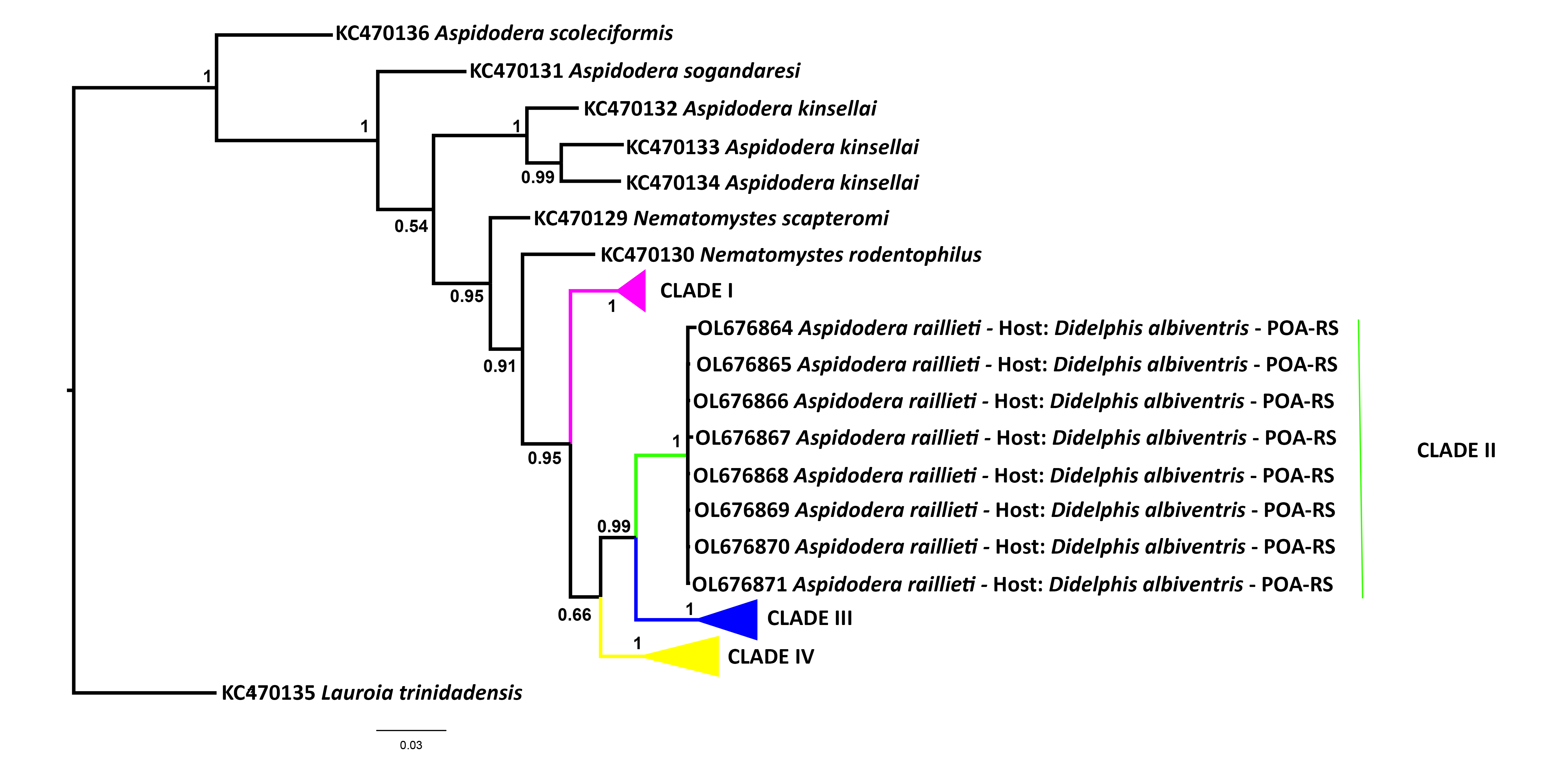

Supplement: Supplementary file 3 — Additional file 3: Figure S2. Bayesian phylogenetic trees of partial MT-CO1 sequences of A. raillieti from this study and of aspidoderid species from GenBank. The sequence of Lauroia trinidadensis was added as an out-group. Clade II of A. raillieti is not collapsed. [file 13071_2022_5288_MOESM3_ESM.tif]
